# RNAseq analysis of bronchial epithelial cells to identify COPD-associated genes and SNPs

**DOI:** 10.1186/s12890-018-0603-y

**Published:** 2018-03-05

**Authors:** Jiyoun Yeo, Diego A. Morales, Tian Chen, Erin L. Crawford, Xiaolu Zhang, Thomas M. Blomquist, Albert M. Levin, Pierre P. Massion, Douglas A. Arenberg, David E. Midthun, Peter J. Mazzone, Steven D. Nathan, Ronald J. Wainz, Patrick Nana-Sinkam, Paige F. S. Willey, Taylor J. Arend, Karanbir Padda, Shuhao Qiu, Alexei Federov, Dawn-Alita R. Hernandez, Jeffrey R. Hammersley, Youngsook Yoon, Fadi Safi, Sadik A. Khuder, James C. Willey

**Affiliations:** 1 Department of Pathology, The University of Toledo College of Medicine, 3000 Arlington Avenue, HEB 219, Toledo, OH 43614 USA; 2 Division of Pulmonary and Critical Care Medicine, Department of Medicine, The University of Toledo College of Medicine, 3000 Arlington Avenue, HEB 219, Toledo, OH 43614 USA; 3 Department of Mathematics and Statistics, The University of Toledo, 2801 W. Bancroft Street, Toledo, OH 43606 USA; 4 Department of Medicine, The University of Toledo College of Medicine, 3000 Arlington Avenue, Toledo, OH 43614 USA; 5 Department of Biostatistics, Henry Ford Health System, 1 Ford Place Detroit, MI, Detroit, MI 48202 USA; 6 Thoracic Program, Vanderbilt Ingram Cancer Center, Nashville, TN 37232 USA; 7 University of Michigan, 500 S. State Street, Ann Arbor, MI 48109 USA; 8 Department of Pulmonary and Critical Care Medicine, Mayo Clinic, 200 1st St SW, Rochester, MN 55905 USA; 9 Department of Pulmonary Medicine, Cleveland Clinic, 9500 Euclid Ave, Cleveland, OH 44195 USA; 10 Department of Pulmonary Medicine, Inova Fairfax Hospital, 3300 Gallows Road, Falls Church, VA 22042-3300 USA; 11 The Toledo Hospital, 2142 N Cove Blvd, Toledo, OH 43606 USA; 12 Division of Pulmonary Diseases and Critical Care Medicine, Virginia Commonwealth University, USA, Richmond, VA 23284-2512 USA; 13 Ohio State University James Comprehensive Cancer Center and Solove Research Institute, Columbus, OH USA; 14 American Enterprise Institute, 1789 Massachusetts Ave NW, Washington, DC 20036 USA; 15 The University of Toledo College of Medicine, 3000 Arlington Avenue, Toledo, OH 43614 USA; 16 Emory University School of Medicine, 1648 Pierce Dr NE, Atlanta, GA 30307 USA; 17 Department of Medicine, The University of Toledo Medical Center, 3000 Arlington Avenue, Toledo, OH 43614 USA; 18 Division of Pulmonary and Critical Care Medicine, Department of Medicine, The University of Toledo College of Medicine, 3000 Arlington Avenue, RHC 0012, Toledo, OH 43614 USA; 19 Division of Pulmonary and Critical Care Medicine, Department of Medicine, The University of Toledo College of Medicine, 3000 Arlington Avenue, Toledo, OH 43614 USA

**Keywords:** COPD, eQTL, *cis*-regulation, GWAS, ERCC5, CAT, CEBPG, GPX1, KEAP1, TP73, XPA, Bronchial epithelial cells

## Abstract

**Background:**

There is a need for more powerful methods to identify low-effect SNPs that contribute to hereditary COPD pathogenesis. We hypothesized that SNPs contributing to COPD risk through *cis*-regulatory effects are enriched in genes comprised by bronchial epithelial cell (BEC) expression patterns associated with COPD.

**Methods:**

To test this hypothesis, normal BEC specimens were obtained by bronchoscopy from 60 subjects: 30 subjects with COPD defined by spirometry (FEV1/FVC < 0.7, FEV1% < 80%), and 30 non-COPD controls. Targeted next generation sequencing was used to measure total and allele-specific expression of 35 genes in genome maintenance (GM) genes pathways linked to COPD pathogenesis, including seven TP53 and CEBP transcription factor family members. Shrinkage linear discriminant analysis (SLDA) was used to identify COPD-classification models. COPD GWAS were queried for putative *cis*-regulatory SNPs in the targeted genes.

**Results:**

On a network basis, TP53 and CEBP transcription factor pathway gene pair network connections, including key DNA repair gene ERCC5, were significantly different in COPD subjects (e.g., Wilcoxon rank sum test for closeness, *p*-value = 5.0E-11). ERCC5 SNP rs4150275 association with chronic bronchitis was identified in a set of Lung Health Study (LHS) COPD GWAS SNPs restricted to those in putative regulatory regions within the targeted genes, and this association was validated in the COPDgene non-hispanic white (NHW) GWAS. ERCC5 SNP rs4150275 is linked (D’ = 1) to ERCC5 SNP rs17655 which displayed differential allelic expression (DAE) in BEC and is an expression quantitative trait locus (eQTL) in lung tissue (*p* = 3.2E-7). SNPs in linkage (D’ = 1) with rs17655 were predicted to alter miRNA binding (rs873601). A classifier model that comprised gene features CAT, CEBPG, GPX1, KEAP1, TP73, and XPA had pooled 10-fold cross-validation receiver operator characteristic area under the curve of 75.4% (95% CI: 66.3%–89.3%). The prevalence of DAE was higher than expected (*p* = 0.0023) in the classifier genes.

**Conclusions:**

GM genes comprised by COPD-associated BEC expression patterns were enriched for SNPs with *cis*-regulatory function, including a putative *cis*-rSNP in ERCC5 that was associated with COPD risk. These findings support additional total and allele-specific expression analysis of gene pathways with high prior likelihood for involvement in COPD pathogenesis.

**Electronic supplementary material:**

The online version of this article (10.1186/s12890-018-0603-y) contains supplementary material, which is available to authorized users.

## Background

Tobacco smoking is the predominant exogenous risk factor for chronic obstructive pulmonary disease (COPD) [1]. However, not all smokers develop COPD, implying that genetic factors contribute to COPD predisposition and pathogenesis. COPD genome-wide association studies (GWAS) have significantly increased our understanding of COPD pathogenesis, yet genomic variants identified through GWAS still explain only a small fraction of hereditary risk [2]. Each variant identified by COPD GWAS required large sample size for detection due to small effect on heritability [3, 4]. Those that remain to be discovered will likely have an even lower effect. To address this challenge, one recent study was designed to discover rare coding variants with large effect on COPD risk, similar to that of alpha-1-antitrypsin deficiency [5]. In another approach, GWAS meta-analyses were used to identify common SNPs with very low effect through very large sample size [3, 6, 7].

The purpose of this study was to identify candidate COPD-risk variants through their role in generation of COPD-associated transcript abundance patterns in bronchial epithelial cells (BEC). BEC coat the surfaces of lung airways and protect lung tissue from the environment. Sub-optimal BEC function is implicated in COPD pathogenesis [8–10]. We hypothesized that hereditary risk for COPD is due to the combined effect of multiple regulatory SNPs that each contribute to generation of BEC transcript abundance patterns associated with hereditary predisposition to COPD. If the proposed hypothesis is correct, a) a study to identify COPD-associated transcript abundance patterns in BEC will have high power and require a relatively small sample size, and b) genes comprised by such patterns will be enriched for *cis*-regulatory (r) SNPs and possibly SNPs significant in COPD GWAS using reduced stringency correction for multiple testing. Recent knowledge regarding predisposition for diseases caused by complex genetics supports this hypothesis in that SNPs associated with COPD and other complex phenotypes are enriched for *cis*- and/or *trans-*regulatory function [11–19]. For example, in a report on the recently updated GTEx database nearly 50% of common GWAS variants of interest were significantly associated with the expression of one or more genes (*P* < 0.05, after correcting for multiple tissue testing) [17].

A common way to identify *cis*-rSNPs is to measure dose effect of alleles at candidate *cis*-rSNPs on *total expression* [17]. SNPs significant by this analysis are referred to as *cis*-expression quantitative trait loci (eQTL). Another way to identify *cis*-rSNPs is by measurement of *allele-specific expression* (ASE) to identify differential allelic expression (DAE) [15, 20, 21]. Importantly, with appropriate methodological conditions, the power to identify *cis*-regulatory SNPs by measurement of ASE is higher than that for measurement of total expression [22], possibly because ASE controls for variation in *trans*-effects, including those resulting from variation in the environment. Importantly, there is a high correlation between *cis*-rSNPs identified by ASE measurement and those identified by as e-QTL by total expression [17]. The recent increase in throughput and reduction in cost of next generation sequencing (NGS) now facilitates large-scale measurement of ASE Tools and best practices for data processing in allelic expression analysis [19, 23].

We used targeted NGS technology to measure total and allele-specific expression (ASE) of 35 selected genome maintenance (GM) genes through RNA sequencing (RNAseq) of BEC RNA from 30 COPD subjects and 30 non-COPD controls. This targeted NGS method employs multiplex competitive PCR-amplicon libraries that provide excellent sequencing depth for all target analytes [24]. This approach is based on RT-PCR technology proven to ensure optimal quality-control characteristics, including high linear dynamic range, signal-to-analyte response, precision, and accuracy, and high correlation with qPCR [24–33].

The targeted NGS RNAseq method used in this study simplified allele-specific transcript abundance measurement and assessment of genes for *cis*-regulatory (r) SNPs manifesting as DAE. Gene expression is well-documented to be a heritable trait [34–36]. Heritable differences in gene expression between individuals are caused by DNA variants that affect the expression level of one allele (*cis*-acting) or both alleles (trans-acting) of a gene [37]. Measurement of DAE is recognized as a powerful approach for identifying *cis*-acting regulatory variation [27, 35, 38]. We applied this technology to assess *cis*-rSNP activity and disease association at the exon level. As previously reported, multivariate exon-level analysis provides a more powerful approach than univariate gene-level analysis for identification of *cis*-rSNPs as well as disease association [39].

The 35 genes selected for analysis have a high prior likelihood for a role in COPD pathogenesis based on studies from this laboratory as well as those from other investigators. They represent antioxidant (AO), DNA repair (DNAR), and cell cycle control (CCC) pathways that play a key role in protecting BEC from inhaled cigarette smoke and toxins from the environment or occupational exposure [8, 25, 27, 29, 30, 40–43]. Importantly, there is significant inter-individual variation in BEC regulation of these key GM pathway genes [30, 40, 44, 45] and many function differently in BEC of COPD subjects compared to matched controls [8, 41–43, 46–54]. For example, genetic variants in CAT, GSTM1, GSTT1, GSTP1, SOD3, NFE2L2, KEAP1, OGG1, XRCC1, XRCC3, XRCC4, XRCC5, CDKN1A, and p53 are reported to be associated with COPD [54–69]. Further, regulation of both CEBP and TP53 family genes as well as many of their targets is different in subjects with COPD [52, 53, 67, 70].

A focus on members of the CEBP transcription factor family (i.e., CEBPA, CEBPD and CEBPG) and TP53 transcription factor family (i.e. TP53, TP63, and TP73) was based on evidence that they play an important role in regulating lung development and differentiation, and regulation of AO, DNAR, and CCC genes in BEC [25, 29, 30, 52, 71–77]. CEBPG is a truncated transcription factor that does not trans-activate but plays a regulatory role through heterodimer formation [78]. CEBPG regulates key GM genes in BEC [25, 29, 30]. CEBPA and CEBPE are anti-proliferative transcription factors that lead to cell differentiation [71, 72, 78]. CEBPB and CEBPD contribute to regulation of cell-cycle progression [73–75]. CEBPG or CEBPA knockout mice die at birth or in adulthood respectively from emphysematous lungs [52, 76], providing experimental confirmation of the important role that sub-optimal function of these regulatory pathways plays in risk for COPD. The role of the TP53 gene transcription factor family in COPD is supported by association of TP53 and CDKN1A alleles with COPD risk and the role of CDKN1A in response to cigarette smoke [66, 67, 79]. TP63 plays a key role in airway epithelial cell proliferation and differentiation [80–88], and is important in maintaining airway epithelial integrity and repair [89]. TP53 and TP73 work together to differentiate BEC into ciliated cells, and TP73 knockout in mice is associated with epithelial cell loss and inflammation of epithelium [90].

## Methods

**Fig. 1:**
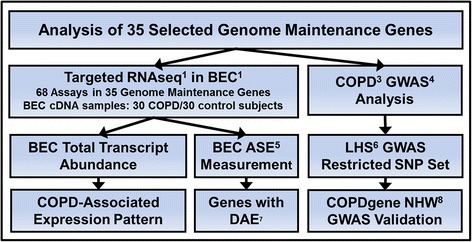
Schematic description of research design. ^1^RNAseq: RNA sequencing by next generation sequencing; ^2^BEC: bronchial epithelial cell; ^3^COPD, chronic obstructive pulmonary disease; ^4^GWAS, genome wide association study; ^5^ASE: allele-specific expression; ^6^LHS GWAS: Lung Health Study Genome Wide Association Study; ^7^DAE: differential allelic expression; ^8^COPDgene NHW: COPDgene Non-Hispanic White Cohort

The goal of this study was to identify BEC gene expression patterns and hereditary DNA variants associated with COPD pathogenesis. Toward this goal we conducted a nested case-control study to a) identify genes associated with COPD based on BEC transcript abundance values and b) assess COPD-associated genes for *cis*-rSNP enrichment, measured as DAE. In parallel, we queried COPD GWAS for significance of putative *cis*-rSNPs in COPD-associated genes. The study design is presented schematically in Fig. 1.

### Study subjects and biospecimens

Homogeneous BEC biospecimens were obtained by bronchoscopic brush biopsy of normal appearing airway (main bronchus) epithelium from 30 COPD and 30 non-COPD control subjects who were enrolled in the Lung Cancer Risk Test (LCRT) study (NCT 01130285 at Clinicaltrials.gov) [91]. The purpose of the LCRT study is to assess clinical validity of the previously reported LCRT to predict risk for lung cancer [25]. LCRT enrollment criteria included high demographic risk for lung cancer (age 50 or more and 20 pack-years smoking or more) and absence of lung cancer at time of enrollment based on chest CT. Summary statistics for the demographic and clinical characteristics of the subjects used in studies presented here are provided in Table 1 and information for each subject are provided in Additional file 1: Table S1 rows 3–12. Additional relevant details of the LCRT study are provided in Additional file 2. In the study presented here, COPD was defined by spirometry as FEV1/FVC < 0.7 and FEV1% expected < 80%. This corresponds to GOLD Stage II-IV COPD [92]. The LCRT enrolled 385 subjects at 11 clinical centers between 2011 and 2013. At each site, BEC were collected into ice-cold normal saline, then pelleted at 300 g and suspended in RNAlater. Biospecimens collected from each LCRT subject were shipped on dry ice overnight to ResearchDx, Irvine, CA, USA) for RNA extraction and storage. All subjects provided written informed consent. Use of tissue samples and corresponding medical/demographic data for this study is approved under UT IRB protocols #108538 and #107844. For this study, COPD and control subjects were selected with a goal to match for age, smoking history, and gender.

**Table 1:** Clinical characteristics of study population

	Non-COPD (*n* = 30)	COPD (*n* = 30)	*p*-value^*†*^
Age, yr	64.3	63.6	0.713
Sex			0.009
Male	11	22	
Female	19	8	
Smoking status			1.0
Current	10	9	
Former	20	21	
Never	0	0	
Pack-years	49	60	0.088
FEV1/FVC	0.81	0.53	5.81E-13
Ethnicity			
White	28	26	
AA	2	4	

^†^*p*-values were calculated using Student’s t-test for age and Pack-years, and Fisher exact test for sex and smoking history

### BEC and peripheral blood cell (PBC) samples for differential allelic expression (DAE) measurement

Reliable measurement of DAE required a sample size large enough to include a sufficient number of heterozygotes. Thus, in addition to the BEC samples and matched PBC samples from 60 LCRT subjects used in COPD classifier development, we evaluated archival BEC (120) and PBC (117) samples from additional subjects who were not characterized for COPD status for the purpose of DAE analysis. Of the total 180 BEC and 177 PBC samples, matched samples were available from 98 subjects.

Summary statistics for the cohort used in allele-specific expression analysis are provided in Additional file 1: Table S2.

### RNA and DNA extraction

For the 60 LCRT subject samples, RNA was extracted from BEC samples at ResearchDx using the RNeasy Kit (Qiagen, Valencia, CA). RNA was treated with DNAse to remove gDNA contamination and assessed for RNA integrity (see below). The RNA was split into two aliquots and frozen at − 80 degrees C. One of the BEC RNA aliquots from each subject was shipped to the University of Toledo where it was first re-tested for gDNA contamination through PCR. Any samples with signal for gDNA were re-treated with DNAse. Samples then were reverse transcribed into cDNA with M-MLV reverse transcriptase (Invitrogen, Carlsbad, CA) using oligo dT primer according to the manufacturer’s protocol. Genomic DNA was extracted from PBC at each clinical site [91], frozen and shipped to the NCI-EDRN bio-specimen bank at NCI-Frederick. One vial of DNA from each subject was provided to this lab for this study. For non-LCRT subject archival samples (120 BEC samples and 117 PBC samples), both BEC RNA and PBC DNA were extracted in this laboratory according to previously described methods [93].

### RNA integrity analysis

RNA extracted from each BEC sample used in this study was assessed for RNA integrity and quantity as previously described [91]. In addition, after careful review of available RNA integrity measurement methods, we chose the 5′/3′ ratio mRNA integrity assay to assess NBEC sample RNA integrity, based on a recent comparison with other methods, including quantitative microfluidic electrophoresis [94]. This method was particularly informative because, since RNA samples were reverse transcribed with poly dT, low quality, fragmented, short length RNA would be associated with lower transcript abundance measured in assays located further away from the 3′ end.

### Competitive multiplex PCR amplicon library preparation

Targeted competitive multiplex PCR amplicon libraries were prepared to quantify total and allele-specific expression at 68 target assays on 35 genes by next generation sequencing (NGS) (in Additional file 1: Table S1) according to previously described methods [24, 26]. Genes with high prior likelihood for association with COPD-risk were selected for analysis based on careful literature review, as described in the background section**.** Included among these was a set of genes previously reported to be relatively unaffected by environmental variation based on cigarette smoking history [30, 40]. *Primers.* We designed a pool of forward and reverse primer sets targeting 68 assays in the 35 genes, using methods described in detail previously [24, 26]. When possible, we measured transcript abundance at multiple sites for each gene because it is not uncommon that probes assessing different alternative transcripts yield different expression patterns due to inter-individual biological variation [28, 95]. For classifier gene DAE analysis, assays were selected from all exonic SNPs with minor allele frequency of > 0.05 identified based on data from the 1000 Genomes Project [96]. Additional primer design methods are provided in Additional file 2*.*

### Internal standards (IS) and internal standard mixture

Methods for preparation of each internal standard and the internal standard mixture were described previously [24, 26]. Details for this study are provided in Additional file 2. Preparation of a PCR amplicon library for each sample involved four sequential PCR reactions, described in Additional file 2. All primer and IS sequences are provided in Additional file 1: Table S1.

### Sequencing

PCR library products were analyzed on an Illumina HiSeq 2500 with TruSeq SBS Kit v4 reagent at Macrogen (Macrogen, Inc., Seoul, South Korea). Macrogen then returned raw sequencing data in FASTQ format. Data Processing, calculation of total or allele-specific target transcript abundance, and filtering to avoid stochastic sampling error were described in detail in [24, 26]. Details for this study are provided in Additional file 2.

### Interaction network differences between COPD cases and control

Bivariate interactions among genes in control or COPD subjects were assessed by Pearson’s correlation. Gene pair network connections were assessed for difference between COPD and control cohorts by Wilcoxon rank sum test for in- and out-degree, betweenness, and closeness, using igraph analysis as described in Additional file 2.

### Test for cis-rSNP enrichment among classifier genes

Inter-individual variation in DAE of a gene is a manifestation of one or more *cis*-rSNPs [15, 17, 19, 22, 23]. To test the question of whether *cis*-rSNPs are enriched in genes with COPD-associated gene expression pattern, we assessed significant difference in prevalence of DAE among COPD-associated genes compared with prevalence of DAE among all genes in multiple different tissues in prior studies [15, 17, 97].

### Lung health study (LHS) and COPDgene non-Hispanic white (NHW) GWAS analysis

Detailed methods for analysis of the LHS dataset (phs000335) and COPDgene dataset phs000765 (NHW) [98, 99] are provided in the Additional file 2.

### Sub-phenotyping of LHS and COPDgene NHW subjects

As presented in Additional file 2: Figure S1 for LHS, prior to analysis the LHS and COPDgene NHW subjects were stratified into Chronic Bronchitis (CB) or Emphysema (EM) sub-phenotypes based on clinical annotation parameters pertaining to chronic productive cough. For LHS, following quality-control analysis a total of 3230 subjects were included. Of these, 527 were chronic bronchitis, 1198 were emphysema, and 1505 were controls. For COPDgene NHW there were a total of 5269 subjects, of which 556 were chronic bronchitis, 2223 were emphysema, and 2490 were controls.

### Statistical analysis

All statistical analyses were performed using R (v 3.2.5) (http://www.R-project.org).

### Shrinkage linear discriminant analysis (SLDA) to develop COPD classifier

BEC transcript abundance values for each assay in each subject were used in development of the COPD classifier model. After filtering, 32 target assays in 23 genes yielded BEC transcript abundance data for reliable total transcript abundance quantification in at least 70% of subjects and were included in statistical analyses. Typically, the cause of low subject representation for an assay was insufficient target molecules loaded into library preparation due to low BEC expression. Missing values were imputed using the corresponding mean value for each assay. Shrinkage Linear Discriminant Analysis (SLDA) was performed to select assays. The overall ranking of each assay was determined by correlation-adjusted t-score (CAT scores) [100]. The 10-fold cross-validation (CV) receiver operating characteristic (ROC) area under the curve (AUC) was applied to identify the best sets of assays, and the pooled 10-fold cross-validated ROC AUC was reported for the selected classifier. For each patient, the classifier assigned a probability score for COPD phenotype. An optimal cut-point to predict the class label based on Youden Index (J) was determined by repeated cross-validation step. Each patient was classified according to optimal cut-point. We compared the model predicted class label to the “true” state of COPD or control subject then calculated the diagnostic odds ratio and confidence interval.

### Inter-individual variation in allele-specific transcript abundance

For each gene, and at each measured transcribed polymorphic locus, we used the F-test to compare inter-individual variation in allelic imbalance in cDNA samples with that in peripheral blood genomic DNA samples. Specifically, gDNA was used as a control because it is expected that every cell will have two copies of gDNA. Therefore, under ordinary circumstances in non-malignant cells, it is expected that the measured ratio between alleles will be close to one, subject to analytical variation. In contrast, inter-individual variation in *cis*-regulation due to polymorphisms may cause inter-individual variation in transcription of one allele to the other. Each allele ratio of read counts was log base 2 transformed prior to further analysis. F-test was performed using R (v 3.2.5) (http://www.R-project.org). GraphPad Prism was used to plot figures.

### Test for cis-rSNP enrichment in COPD classifier genes

We measured DAE as an indicator that a gene contained one or more *cis*-rSNPs. To assess for *cis*-rSNP enrichment among classifier genes, we compared the fraction of genes with DAE among the genes comprised by the classifier to the fraction of genes with DAE in a large prior study [15, 101] using the N-1 Chi-Squared test [102] at MEDCALC (https://www.medcalc.org/calc/comparison_of_proportions.php).

### Bivariate analysis

We assessed difference in inter-gene correlation of log-transformed transcript abundance by Pearson correlation coefficient (*r*-value). We used the Fisher r-to-z transformation (Z-score) to assess the significance of difference between two correlation coefficients in two groups.

### Covariate analysis

Analysis of covariance (ANCOVA) was used to assess COPD vs control group difference in transcript abundance after controlling for single covariates.

### Correction for multiple testing

We used Bonferroni adjustment to correct for multiple testing in GWAS analysis. GTEx data regarding eQTL in lung tissue are reported with the *p*-values corrected for multiple testing and calculated by GTEx. We report analyses of individual gene-pair correlation changes between control and COPD and analyses of differences in means after correction for covariates (ANCOVA) without correction for multiple testing. This is justified because each of the features tested was selected for analysis based on prior association with COPD, thereby reducing likelihood of false discovery.

## Results

### RNA and DNA samples

RNA extracted from each BEC sample met previously described thresholds for RNA integrity and quantity as described in methods [91].

### Targeted RNAseq expression data

Total transcript abundance values meeting the QC threshold were obtained in BEC samples from at least 70% of subjects for 32 target assays in 23 genes and this set was used in univariate and multivariate analyses.

### Interaction network differences between COPD cases and control

**Fig. 2:**
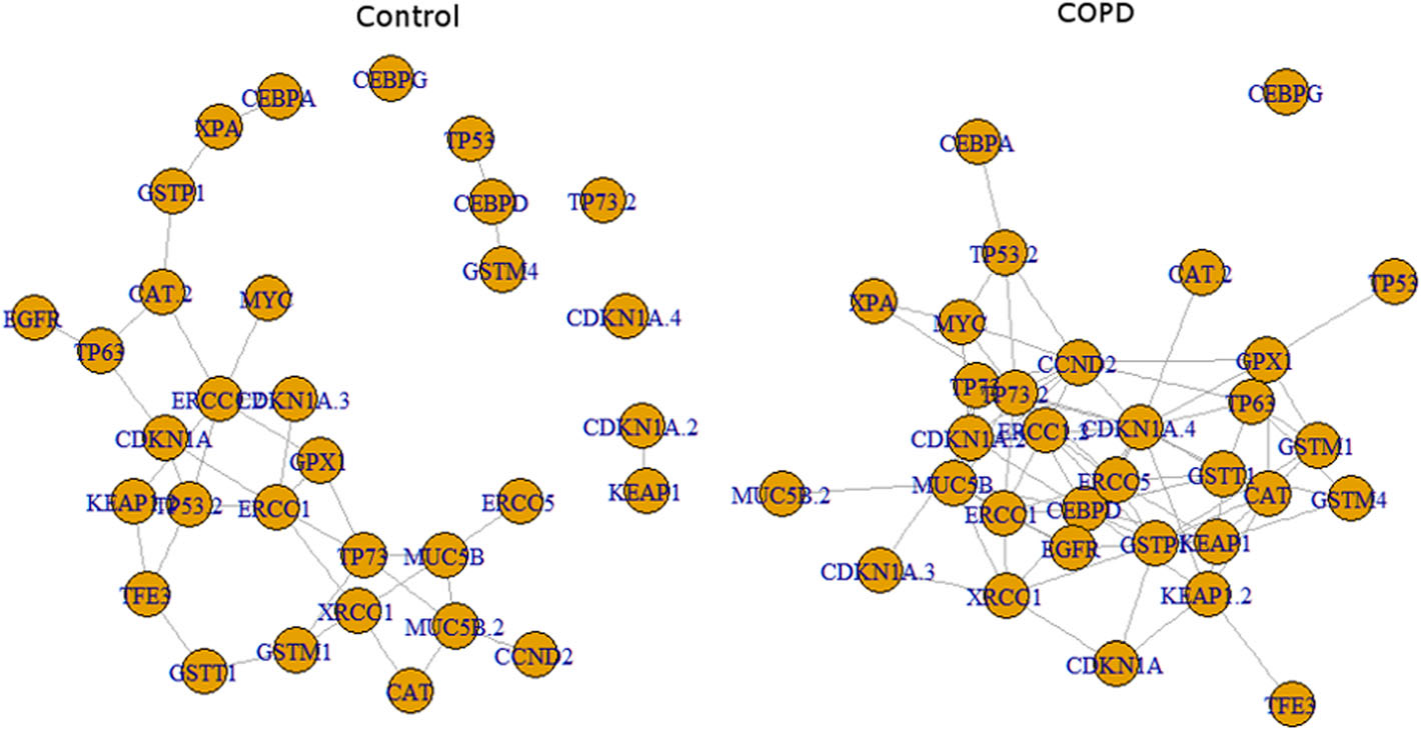
Network of bivariate correlation among genes (transcript abundance values) for control and COPD cohorts. Each line represents Pearson *r*-value with *p*-value < 0.05. Left: Control, Right: COPD. (See Additional file 1: Table S3 for r- and *p*-value of each gene pair)

**Fig. 3:**
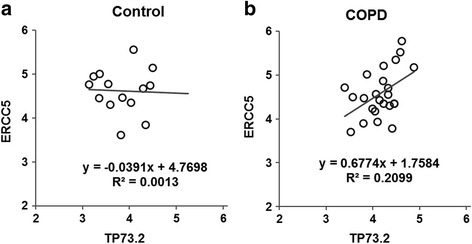
Inter-gene correlation differences in control vs COPD cohorts. **a**, **b** TP73–2 vs ERCC5

On a network basis, TP53 and CEBP transcription factor pathway gene pair network connections were different between COPD and control cohorts as measured by Wilcoxon rank sum test for in- and out-degree (*p*-value = 7.0E-05), betweenness (*p*-value = 0.00437), and closeness (*p*-value = 5.0E-11) (Fig. 2). Consistent with this, the total number of inter-gene correlation connections (lines) among the tested assays was higher among COPD individuals. Inter-gene correlation data in relationship to transcription factors are presented in Additional file 1: Table S3. Notably, ERCC5 was more highly correlated with both CEBPD and TP73 in COPD compared with controls **(**Fig. 3**,** Additional file 1: Table S3). In contrast, CEPBD correlation with TP53 was decreased in COPD.

### Analysis of covariance (ANCOVA)

After adjustment for covariate effects, there was a difference in mean expression between COPD and controls for several classifier genes. Specifically CEBPG, GPX1, and TP73 were expressed at a higher level in COPD compared to controls while KEAP1 was expressed at a lower level (Additional file 1: Table S4).

### Identification of genes with COPD-associated expression pattern

**Fig. 4:**
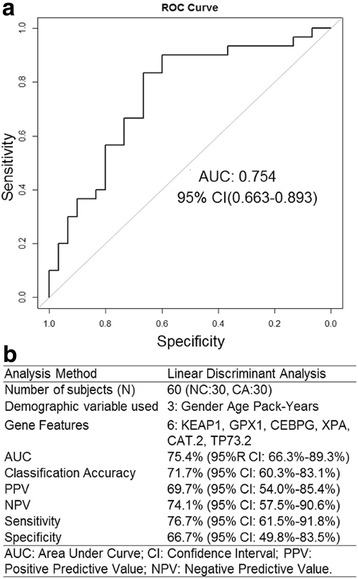
Receiver operating characteristic curve (ROC) (**a**) and summary of performance of classifier (**b**) in 30 control and 30 COPD subjects

SLDA was used to rank each feature for classification ability according to correlation-adjusted t (CAT)-score (See Additional file 1: Table S1). After 10-fold cross-validation, the classifier with best ROC AUC comprised nine features, including the three demographic variables sex, age, and smoking history in pack-years, and six genes: CAT, CEBPG, GPX1, KEAP1, TP73, and XPA **(**Fig. 4**,** Table 2**,** and Additional file 1: Table S1 rows 14–19). The 10-fold cross-validation AUC for the model was 75.4% (95% CI: 66.3%–89.3%). As reported above, ERCC5 has altered correlation with transcription factor TP73 comprised by the classifier **(**Fig. 3**)** and other genes as measured by iGraph **(**Fig. 2**).**

**Table 2:** COPD classifier gene features selected by SLDA^1^ and 10-fold cross-validation

Feature	Gene Function	CAT^2^ score	Ranking	Missing value (%)
KEAP1	AO^3^	3.35	1	8%
GPX1	AO	3.32	2	5%
CEBPG	TF^4^	2.98	3	18%
XPA	DNAR^5^	2.64	4	28%
CAT-2	AO	2.64	5	22%
TP73–2	CCC^6^/DNAR	2.30	6	28%

^1^*SLDA* shrinkage linear discriminant analysis, ^2^*CAT score* correlation-adjusted t-scores, ^3^*AO* antioxidant, ^4^*TF* transcription factor, ^5^*DNAR* DNA repair, ^6^*CCC* cell cycle control

### Query of LHS GWAS for targeted genes

Details of LHS GWAS analysis are provided in Additional file 2: Figure S1). Briefly, the LHS COPD GWAS subjects were stratified into chronic bronchitis (CB) or emphysema (EM) sub-phenotypes. All LHS SNPs that passed quality control were restricted to putative *cis*-regulatory regions. Then, SNPs in this restricted set with *p* < 0.05 for association with LHS CB or LHS EM subjects were identified. The resulting SNP sets were designated {LHS CB Restricted} and {LHS EM Restricted}.

### Integration of restricted LHS SNP sets with genes targeted in this study

The intersection of the 35 genes targeted in this study with the {LHS CB Restricted} SNP set comprised six linked SNPs in ERCC5, each with *P* < 0.05 (Additional file 1: Table S5). In contrast, no SNPs were identified in the intersection of the 35 targeted genes and the {LHS EM Restricted} SNP set.

### Validation of ERCC5 SNP association with chronic bronchitis in the COPDgene cohort

The independent COPDgene NHW phs000765 cohort [3, 98] was stratified according to CB or EM sub-phenotype using the same criteria used to sub-phenotype LHS. COPDgene NHW CB and COPDgene NHW EM were each queried for rs4150275**,** which was chosen to represent the 6 linked SNPs (Additional file 2: Figure S2). In this validation test, because one SNP was queried in two sub-phenotypes, the Bonferonni adjusted threshold for significance was a = 0.05/2 = 0.025. As with LHS analysis, rs4150275 was significant in COPDgene NHW CB (*p* = 0.0046) but not COPDgene NHW EM. Importantly, the haplotype represented by rs4150275 allele A was associated with CB in both LHS and COPDgene NHW.

### Assessment of genes with COPD-associated RNAseq patterns for DAE in BEC

ERCC5 displays inter-individual variation in DAE in BEC, measured at multiple SNPs, including rs17655, which is linked (D’) to rs4150275) [93]. Further, rs873601 which is linked to rs17655 is predicted to alter miRNA binding sites and likely plays a key functional *cis*-regulatory role [103].

**Fig. 5:**
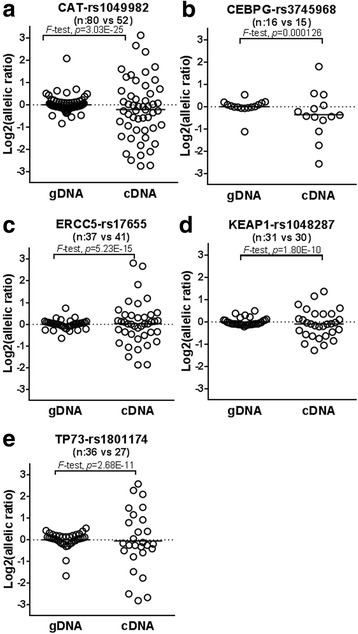
Inter-individual variation allelic ratio for cDNA compared with gDNA. Each symbol represents results from a single heterozygous individual. **a** CAT-rs1049982, **b** CEBPG-rs3745968, **c** ERCC5-rs17655, **d** KEAP1-rs1048287, **e** TP73-rs1801174

We identified at least one expressed SNP with MAF > 0.05 in four of the six COPD classifier genes selected by SLDA; CAT: rs1049982, CEBPG: rs3745968, KEAP1:rs1048287, and TP73:rs1801174. For each of these SNPs, the number of heterozygotes among the cDNA samples was close to Hardy-Weinberg Equilibrium expectations (Table 3) and comparable to that observed among gDNA samples. Inter-individual variation in allelic-imbalance in cDNA was significantly higher (p < 0.05) than that in gDNA at each of these four sites after Bonferroni adjustment for multiple testing (Table 3, Fig. 5). The rate of DAE among ERCC5 and the four measurable classifier genes was 100% (4/4) which was significantly higher (*p* = 0.0023) than the 30% of genes that demonstrated DAE in lung tissue (5884/19,725) [17] or lymphoblastoid cell lines (2935/9751) [101].

**Table 3:** SLDA COPD classifier gene differential allelic expression (DAE) in bronchial epithelial cell (BEC) or in GTEx lung tissue database

		DAE in BEC			GTEx Lung Tissue (*n* = 278)	
^1^SNP	MAF	Subjects Assessed (*n*)	^2^Heterozygote Subjects with DAE data (*n*)	^3^*p-*value	eQTL	^4^*p-*value
KEAP1-rs1048287	0.1	159	30	*9.02E-10*	KEAP1	^5^N.R.
CEBPG-rs3745968	0.11	128	17	*6.35E-04*	CEBPG	N.R.
CAT-rs1049982	0.34	156	52	*1.51E-24*	CAT	*1.95E-08*
TP73-rs1801174	0.09	158	27	*1.34E-10*	TP73	N.R.

Significant *p*-values indicated in italicized font

^1^SNP that served as marker for DAE. SNPs with highest minor allele frequency chosen

^2^*n* = number of subjects for whom each SNP allele was measurable in BEC after filtering to prevent stochastic sampling error. The fraction of gDNA samples with heterozygotes was comparable to that for cDNA samples and both approximated Hardy Weinberg Equilibrium expectations

^3^*p*-value for F-test comparing inter-individual variation in cDNA to inter-individual variation in gDNA samples

^4^p-value reported in GTEx database

^5^*N.R* not reported

### Assessment of BEC putative cis-rSNPs for lung tissue quantitative trait loci (eQTL) status in GTEx database

We queried the Genotype-Tissue Expression (GTEx) database [17, 104] for lung tissue eQTL at SNPs in that were associated with DAE in BEC, including classifier genes CAT, CEBPG, GPX1, and TP73 **(**Table 3**)**, and ERCC5. GTEx measured eQTL as dose-effect of genotype on total transcript abundance**.** SNPs associated with lung tissue eQTL include rs17655 (*p* = 3.2E-7), located in the 3′ untranslated region of ERCC5, and rs1049982 (*p* = 1.95E-08), located in the promoter of CAT. Notably, for each of these SNPs, the same allele associated with higher expression in GTEx lung tissue was significantly more likely to be expressed at higher level than the opposite allele in BEC. SNPs associated with BEC DAE in the other genes were not associated with significant eQTL in lung tissue in GTEx **(**Table 3**)**.

## Discussion

Data reported here support the hypothesis that low-effect COPD risk/pathogenesis SNPs may be discovered through enrichment as *cis*-regulatory SNPs in genes that display COPD-associated BEC expression patterns. Moreover, by integrating the BEC RNAseq data with COPD GWAS [98, 99], ENCODE [105], and GTEx [17, 104] databases, we identified ERCC5 SNP rs873601 as a plausible functional connection between ERCC5 DAE (measured at rs17655), and association of rs4150275 with chronic bronchitis in COPD GWAS (Additional file 1: Table S6). [103, 106]. Thus, these data support the role of rs873601 in ERCC5 *cis*-regulation associated with COPD pathogenesis and risk. That said, other SNPs linked to rs4150275 also are predicted to affect binding of transacting proteins and also could play a role (Additional file 1: Table S5).

### Putative cis-regulatory SNPs in SLDA classifier genes

CAT SNP rs1049982 is predicted to have *cis*-regulatory function because it is in the 5′ untranslated region near the promoter and affects binding of POLR2A. In addition, this SNP was identified as a lung tissue eQTL in lung tissue [17, 104]. Based on these characteristics, this SNP would be a suitable target for experimental confirmation of function in BEC [107]. With respect to CEBPG, KEAP1, and TP73 it is likely that SNPs other than those used to measure DAE in this study are responsible for *cis*-regulation of these genes in BEC and additional experimental studies will be necessary to answer this question.

COPD-associated SNPs have been reported for several of the classifier genes (see background section) but, to our knowledge, not validated in individual GWAS. It will be worthwhile to assess recently completed COPD meta-analysis GWAS for SNPs in these genes using reduced stringency for false reporting.

### Effect of study design characteristics selected to optimize power

Several methodological approaches were implemented in this study to maximize the power to identify BEC transcription patterns associated with COPD. For example, genes with high prior likelihood for hereditary COPD-risk association were targeted. In addition, a transcript abundance measurement platform with excellent analytical performance characteristics was used. Specifically, there was no measurable signal-to-analyte compression with the method used in this study [25, 28] and the targeted PCR method used resulted in abundant signal for each analyte. This resulted in robust collection of data from each specimen, comparing favorably with other methods, such as microarrays or whole exome sequencing analysis [8, 17, 41]. Moreover, analysis of cell populations homogeneous for a particular cell type, such as the homogeneous BEC population samples in this study, increases the power to identify disease-associated transcript abundance patterns and eQTL [95, 108, 109]. According to ASE measurement by targeted RNAseq, DAE was clearly detected for marker SNPs in classifier genes CEBPG, KEAP1, and TP73 in BEC **(**Table 3**)**. However, lung tissue eQTL was not reported for any SNPs in these genes measured by whole transcriptome RNAseq in GTEx study [17]. This observation is likely due to difference in specimen type (i.e. homogeneous BEC vs heterogeneous lung tissue), and deeper coverage obtained by targeted RNAseq for ASE in this study.

### Limits of study and opportunities

It is evident that COPD risk SNPs discovered in the future will have low effect because they are very rare and/or they are common but their individual contribution to risk is low. Results presented here exemplify both the challenge to identify low-effect complex disease risk variants and the opportunity of the approach used. The low effect of rs4150275 is likely due to multiple factors. For example, the rs4150275 A allele prevalence in European populations is 5%. Moreover, rs873601, the putative functional SNP linked to DAE SNP rs17655 and COPD risk SNP rs4150275, may be one of multiple SNPs that contribute to ERCC5 transcription regulation [93]. As such, there is a need to directly measure function of putative *cis*-regulatory SNPs through recently developed high throughput NGS methods [110–112]. Another factor contributing to low effect of rs4150275 is that, based on data presented here, ERCC5 is likely one of many genes that contribute to COPD risk when sub-optimally regulated. In this study we increased the power to identify COPD risk SNPs with *cis*-regulatory function in BEC by studying homogeneous BEC biospecimens. It is likely that hereditary variants affecting gene expression in lung fibroblasts and immune cells also contribute to COPD predisposition. Thus, homogeneous populations of these cell types should be included in future studies.

## Conclusion

We report that low-effect COPD risk SNPs may be identified through enrichment as *cis*-regulatory SNPs in genes that display COPD-associated BEC expression patterns. These findings support broader application of the approach presented here, including further targeted RNAseq analysis of BEC and homogeneous populations of other lung cell types to identify COPD associated expression patterns and *cis*-rSNPs in genes comprised by the expression patterns, followed by test of associated *cis*-rSNPs in large GWAS meta-analyses. This approach promises to facilitate progress toward the important goal of identifying a set of COPD risk variants with sufficient effect on COPD pathogenesis and variation in hereditary risk to have clinical utility. This knowledge is expected to lead to better COPD prevention and treatment strategies.

## Additional files

Additional file 1: Tables S1, S2, S3, S4, S5 and S6. **Table S1.** This table provides: a) Gene-specific assay information including SNP sites, primer and internal standard sequences, b) Subject-specific demographic information, and c) assay- and subject-specific transcript abundance values (target gene molecules/10^6^ ACTB molecules). **Table S2.** Population used for allele specific expression analysis: Summary demographic characteristics of the study population of allele specific expression (subject total *n* = 180). **Table S3.** Transcription factor-target inter-gene correlation in Control, COPD, or All subjects (*p*-value < 0.05). **Table S4.** Analysis of covariance (ANCOVA). Gene expression values (Independent Variables) significantly correlated (positively or negatively) with COPD subjects (Dependent Variable) after control for expression values of other genes (Covariates). **Table S5.** ERCC5 SNPs linked to rs17655 and rs873601 (D > 0.95) and with *p* < 0.05 in LHS and COPDgene NHW CB cohorts. COPD GWAS *p*-values, population-specific genotype frequencies, and epigenetic annotation information from Haploreg/Encode. **Table S6.** Haplotype structure between COPDgene NHW^1^ associated SNP rs4150275, putative functional cis-rSNP rs873601, and DAE^2^ SNP rs17655. (XLSX 109 kb)

Additional file 2: Supplementary Methods, and Figures S1 and S2. Supplementary Methods: Study subjects and tissues, RNA and DNA extraction, preparation of internal standard mixture (ISM) primers, PCR steps, sequencing, data processing and calculation of total or allele-specific target transcript abundance, filtering against stochastic sampling error, Measurement of inter-individual variation in allele-specific transcript abundance, Assessment of BEC eQTL in publically available Genotype-Tissue Expression (GTEx) database, GWAS analysis, sub-phenotyping of LHS and COPDgene NHW subjects, restriction of LHS GWAS SNP set, and integration of COPD-associated putative cis-rSNPs with GWAS. **Figure S1.** LHS Sub-phenotyping and SNP Restriction. **Figure S2.** Integration of 35 targeted genes with {LHS CB Restricted} and {LHS EM Restricted} SNP sets, followed by test for validation in the COPDgene NHW CB and EM cohorts. (DOCX 93 kb)

## Abbreviations

ANCOVA: Analysis of covariance
ASE: Allele specific expression
AUC: Area under the curve
BEC: Bronchial epithelial cell
CAT: Correlation adjusted t
CB: Chronic bronchitis
cis-rSNP: *cis*-regulatory single nucleotide polymorphism
COPD: Chronic obstructive pulmonary disease
DAE: Differential allelic expression
EM: Emphysema
eQTL: Expression quantitative trait loci
GM: Genome maintenance
GTEX: Genotype-Tissue Expression
GWAS: Genome-wide association studies
IRB: Institutional Review Board
IS: Internal standard
LCRT: Lung cancer risk test
LHS: Lung health study
NGS: Next generation sequencing
NHW: Non-Hispanic white
ROC: Receiver Operator Characteristic
SLDA: Shrinkage linear discriminant analysis
SNP: Single nucleotide polymorphism
