# Supplementary material for: RNAseq analysis of bronchial epithelial cells to identify COPD-associated genes and SNPs
Source: BMC Pulm Med. 2018 Mar 5;18:42. doi: 10.1186/s12890-018-0603-y (PMC5838965; doi:10.1186/s12890-018-0603-y)
Supplement: Supplementary file 2 — Supplementary Methods, and Figures S1 and S2. Supplementary Methods: Study subjects and tissues, RNA and DNA extraction, preparation of internal standard mixture (ISM) primers, PCR steps, sequencing, data processing and calculation of total or allele-specific target transcript abundance, filtering against stochastic sampling error, Measurement of inter-individual variation in allele-specific transcript abundance, Assessment of BEC eQTL in publically available Genotype-Tissue Expression (GTEx) database, GWAS analysis, sub-phenotyping of LHS and COPDgene NHW subjects, restriction of LHS GWAS SNP set, and integration of COPD-associated putative cis-rSNPs with GWAS. Figure S1. LHS Sub-phenotyping and SNP Restriction. Figure S2. Integration of 35 targeted genes with {LHS CB Restricted} and {LHS EM Restricted} SNP sets, followed by test for validation in the COPDgene NHW CB and EM cohorts. (DOCX 93 kb) [file 12890_2018_603_MOESM2_ESM.docx]

**RNAseq analysis of bronchial epithelial cells to identify COPD associated genes and SNPs**

Jiyoun Yeo, Diego A. Morales, Tian Chen, Erin L. Crawford, Xiaolu Zhang, Thomas M. Blomquist, Albert M. Levin, Pierre P. Massion, Douglas. A. Arenberg, David E. Midthun, Peter. J. Mazzone, Steven D. Nathan, Ronald J. Wainz, Patrick Nana-Sinkam, Paige F.S. Willey, Taylor J. Arend, Karanbir Padda, Shuhao Qiu, Alexei Federov, Dawn-Alita R. Hernandez, Jeffrey R. Hammersley, Youngsook Yoon, Fadi Safi, Sadik A Khuder, James C. Willey

**ONLINE DATA SUPPLEMENT**

**Supplementary Methods**

Study subjects and tissues

*BEC samples for identification of of COPD-associated gene expression.* Normal BEC samples were obtained by bronchoscopic brush biopsy of normal appearing bronchial epithelium from 30 COPD subjects and 30 non-COPD control subjects who were enrolled into the Lung Cancer Risk Test (LCRT) study (Clinicaltrials.gov) [1]. LCRT enrollment criteria included high demographic risk for lung cancer (age 50 or more and 20 pack-years smoking or more) and absence of lung cancer at time of enrollment based on chest CT. COPD was defined by spirometry as FEV1/FVC <0.7 and FEV1 % expected < 80%. The LCRT enrolled 385 subjects at 11 clinical centers between 2011 and 2013. At each site, BEC were collected into ice-cold normal saline, then pelleted at 300g and suspended in RNAlater. Biospecimens collected from each LCRT subject were shipped on dry ice overnight to ResearchDx, Irvine, CA, USA) for RNA extraction and storage. The purpose of the LCRT study is to assess clinical validity of the previously reported LCRT to predict risk for lung cancer [2]. All subjects provided written informed consent. Use of tissue samples and corresponding medical/demographic data for this study is approved under UT IRB protocols #108538 and #107844. For this study, COPD and control subjects were selected with a goal to match for age, smoking history, and gender.

*BEC and Peripheral Blood Cell (PBC) samples for Differential Allelic Expression (DAE) measurement.* Reliable measurement of DAE required a sample size large enough to include a sufficient number of heterozygotes. Thus, in addition to the BEC samples and matched PBC samples from 60 LCRT subjects used in COPD classifier development, we evaluated archival BEC and PBC samples from an additional 120 subjects and 117 subjects, respectively, for the purpose of DAE analysis. Of the total 180 BEC and 177 PBC samples, matched samples were available from 98 subjects.

RNA and DNA extraction

For the 60 LCRT subject samples, RNA was extracted from BEC samples at ResearchDx using the RNeasy Kit (Qiagen, Valencia, CA). RNA was treated with DNAse to remove gDNA contamination. The RNA was split into two aliquots and frozen at -80 degrees C. One of the BEC RNA aliquots from each subject was shipped to the University of Toledo where it was first re-tested for gDNA contamination through PCR. Any samples with signal for gDNA were re-treated with DNAse. Samples then were reverse transcribed into cDNA with M-MLV reverse transcriptase (Invitrogen, Carlsbad, CA) using oligo dT primer according to the manufacturer’s protocol. Genomic DNA was extracted from PBC at each clinical site [1], frozen and shipped to the NCI-EDRN bio-specimen bank at NCI-Frederick. One vial of DNA from each subject was provided to this lab for this study. For non-LCRT subject archival samples (120 BEC samples and 117 PBC samples), both BEC RNA and PBC DNA were extracted in this laboratory according to previously described methods [3].

*Primers*

We designed a pool of forward and reverse primer sets targeting 68 assays in the 35 genes. For each gene, all exonic SNPs with minor allele frequency of >0.05 were identified based on data from the 1,000 genomes project [4]. Then, primers were designed to target a 101-bp region that spanned each of up to four unlinked (i.e., r^2^ <0.02) SNPs. For some genes (e.g., CEBPD, GSTP1, XPA) there were no exonic SNPs with MAF >0.05, so the SNP with highest MAF was selected. Primers then were synthesized by Integrated DNA Technologies (IDT, Coralville, Iowa, USA). A universal tail sequence (arrayed primer extension: APEX-2) [5] that is not present in the human genome was added to the 5’ end of each target gene primer and served as a linker for the barcoding PCR step (PCR 3). For PCR 3, primers were designed with APEX sequence at the 3’ end, barcode sequences in the middle, and Illumina read 1 or read 2 sequences at each 5’ end. A combination of forward and reverse barcodes served to distinguish subjects. Read 1 and read 2 sequences also served as linkers for the last step PCR (PCR 4) that added a platform specific adaptor for the Illumina sequencing flow cell.

*Preparation of internal standard mixture (ISM)*

For each assay, we designed an internal standard (IS) with 6 bases alteration from the reference DNA (NCBI, GRCh37) and each IS was produced as a single-stranded product through batch synthesis (CustomArray. Inc, Bothell, WA, USA). We then isolated each IS as a full-length, double stranded product in separate PCR-amplification reactions with target-specific primers (without APEX-2 sequence) and quantified each PCR product on a Bioanalyzer 2100. The IS PCR product for each target gene, but not the loading control reference gene (ACTB) IS, was mixed at 1:1 ratio, and purified on agarose gel. The 101 bp band was isolated from agarose using the QIAX II gel extraction kit (Qiagen, Valencia, CA). This purified 1:1 IS mixture was quantified then combined with a known quantity of purified IS for the ACTB loading control gene target to create an internal standards mixture (ISM) for use in PCR. The ISM comprised each target IS at a concentration of 10^-15^M (600 copies/μl) and the ACTB IS at concentration of 10^-14^M (6,000 copies/μl).

*PCR 1*. *70-plex PCR of each sample*. For each reaction, a 10 µL reaction volume was prepared containing 1 µL cDNA sample, 1 µL ISM, 1 µL 70-plex primer pool containing for each target specific forward (F) and reverse (R) primers with APEX-2 linker tail (100 nM each), 1 µL of 2 mM dNTPs, 1 µL of 10× Idaho Technology reaction buffer with 30 mM MgCl_2_, 0.1 µL of Promega GoTaq Hot Start Taq polymerase (5 U/uL), and 4.9 µL of RNase-free water. Each reaction mixture was amplified using an air thermal cycler (RapidCycler; Idaho Technology, Inc. Idaho Falls, Idaho). After an initial Taq activation at 95°C for 3 min, the reaction mixture was incubated for 20 cycles, with each cycle consisting of 94°C for 5 sec (denaturing), 58°C for 10 sec (annealing), and 72°C for 15 sec (extension).

*PCR 2: Microfluidic PCR of each sample.* For each sample, the PCR 1 product was column-purified (50uL elution, QIAquick® PCR Purification Kit, Qiagen) to remove residual primers and salts, then 1µL of purified 1^st^ step PCR product was loaded into one of 48 sample wells of a Fluidigm AccessArray™ chip (South San Francisco, CA, USA). Primer pairs for one or two assays were loaded into each of the 48 primer wells on the chip. The AccessArray™ then combined aliquots from each of the 48 sample wells with aliquots from each of the 48 primer wells (total 70 sets of primers), and the resulting 2,304 reaction mixtures were PCR- amplified in the microfluidic chambers according to AccessArray™ protocol. The 70-PCR products for each sample were harvested back into their respective sample wells. After completion of PCR, we checked PCR products randomly with the Bioanalyzer to ensure presence of correct size PCR products.

*PCR 3: Barcoding of each sample.* In PCR 3, the PCR 2 product was column-purified then one microliter of purified product from each sample was used for barcoding PCR. A 10 µL PCR reaction volume was prepared containing: 1 µL of purified PCR 2 product, 1µL of 10 µM each forward and reverse barcoding primer, 1 µL of 2 mM dNTPs, 1 µL of 10× Idaho Technology reaction buffer with 30 mM MgCl_2_, 0.1 µL of Promega GoTaq Hot Start Taq polymerase (5 u/µL) and 4.9 µL of RNase-free water. Each barcoding was done with an air thermal cycler under the following conditions: 95°C 3 min (Taq activation); 15 cycles of 94°C 5 sec (denaturation), 58°C 10 sec (annealing), and 72°C 15 sec (extension). We randomly checked PCR products with the Bioanalyzer 2100 to ensure presence of correct size PCR products.

*PCR 4: Addition of platform adapters*. For each PCR 4 reaction to add platform-specific sequences to each barcoded product, a 10 µL reaction volume was prepared containing: 1 µL of PCR 3 product that was 100-fold diluted with TE (1/100-fold, TE), 1µL of 10 µM P5 and P7 primer mixture each, 1 µL of 2 mM dNTPs, 1 µL of 10× Idaho Technology reaction buffer with 30 mM MgCl_2_, 0.1 µL of Promega GoTaq Hot Start Taq polymerase (5 u/µL) and 5.9 µL of RNase-free water. Each sample was cycled in the air thermal cycler separately under the same PCR conditions used for PCR 3. After checking peaks of PCR products and concentration randomly, products from all 60 samples were combined at equal volumes and then purified on QIAquick PCR purification kit (Qiagen, Limburg). The purified products were checked for concentration by the Bioanlayzer 2100 and then sent for sequencing.

Sequencing

The PCR library with products for 68 gene targets from 30 COPD and 30 control BEC samples was sent to Macrogen (macrogen.com) for Illumina Hiseq 2500 with TruSeq SBS Kit v4 reagent. Macrogen then returned raw sequencing data in FASTQ format.

Data Processing and calculation of total or allele-specific target transcript abundance

Read 1 and Read 2 sequences were joined and then de-multiplexed based on dual-index barcoding on each template. The “captured” region between the primer sequences was aligned using custom alignment with Approximate String matching algorithm as previous described [6]. For each cDNA sample, the “counts” for aligned sequences for each allele of native template (NT) or competitive IS for each target assay and for the ACTB loading control gene assay, were displayed in an excel file.

For each cDNA sample, calculation of NT molecules for the loading control gene assay (ACTB) and each target gene assay was based on a known number of input IS molecules in 1µL of ISM in reaction mixture (i.e., ACTB 6000 molecules and each target 600 molecules) [6]. NT/IS ratio for each assay was calculated based on comparison of read counts for each NT to respective IS. Then, each NT/IS ratio was multiplied times the known molecules of input IS. Last, the transcript abundance value normalized for loading was calculated for each target assay (target gene molecules/10^6^ ACTB molecules).

Filtering against stochastic sampling error

Stochastic sampling increases analytical variation in measurement of transcript abundance as the number of target molecules loaded into the library preparation, and/or the number of library product molecules loaded into the sequencer decreases below a certain threshold [7]. We recently developed a filter in the pipeline that considers a threshold minimum molecules added to library preparation and a threshold minimum number of sequencing read counts to prevent unacceptable analytical variation due to stochastic sampling [7]. This filter is consistent with another recently described approach for analysis of RNAseq data to measure ASE [8].

Measurement of inter-individual variation in allele-specific transcript abundance

Allelic imbalance is measurable only in heterozygotic subjects. After identifying genes that contributed to the best COPD classifier, we identified SNPs within the coding region of each of the classifier genes with sufficiently high minor allele frequency (MAF) to enable identification of the number of heterozygotes necessary to reach power with the number of subjects available for this study. It was possible to identify SNPs with sufficiently high MAF in four of the classifier genes (CAT, CEBPG, KEAP2, and TP73) and ERCC5 but not in GPX1, or XPA. Assays with a minimum of eight heterozygous subjects for both cDNA and gDNA after filtering for low library copies and/or sequence counts, were included in analysis by F-test.

Assessment of BEC eQTL in publically available Genotype-Tissue Expression (GTEx) database

ERCC5 SNP rs17655 and each COPD classifier gene SNP that displayed significant *cis*-rSNP function in BEC in the form of DAE were assessed for eQTL in the GTEx lung tissue database[9] [10]. The GTEx project used whole transcriptome RNAseq analysis to measure genome-wide lung tissue total transcript abundance and SNPchip to genotype each tissue biospecimen, then assessed correlation of transcript abundance with genotype at each measured SNP. Each classifier gene SNP that demonstrated *cis*-rSNP activity in the form of DAE in BEC in our studies was queried for eQTL function in lung tissue in the GTEx database [9] [10]. A Bonferroni adjusted cut-off *p*-value of 0.01 (0.05/5 SNPs assessed) was used as threshold for significance.

GWAS Analysis

LHS dataset (phs000335) and COPDgene dataset phs000765 (NHW) were each downloaded from the database of Genotypes and Phenotypes (dbGaP) through approved project #200623 and UT IRB protocol #7828. Each dataset contained information on two COPD sub-phenotypes: Chronic Bronchitis (CB) and Emphysema (E). We used PLINK v1.9 [11] to perform quality control analysis and to create genetic scores. Principal Component Analysis (PCA) using Eigenstrat Algorithm [12] was performed as a population check, using the 1000 Genomes Project Dataset as a reference. We oriented alleles relative to the 1000 genomes dataset, and gave each allele equal weight. After confirming ethnicity reported in the respective databases and orienting alleles, linked SNPs were imputed using The University of Michigan’s Imputation Server, which used Minimac3. Minimac3 is a low memory, computationally efficient implementation of a Markov Chain Haplotyping algorithm (MaCH). The Markov Chain would be used as a stochastic model to describe a sequence of possible alleles in which the probability of each allele is solely dependent on the previously determined allele. The parameters used for imputation were as follows: 1000 G Phase 3v5, SHAPEIT Phasing (for unphased input files), mixed population and mode: quality control and imputations. These parameters were chosen in order to work with a commonly accepted 1000 genomes reference dataset. We then used PLINKv1.9 to create genetic scores through logistic regression analysis and annotated the data. Calculation of bivariate SNP paired allele frequencies was done according to previously described method using PLINK [11].

Sub-phenotyping of LHS and COPDgene NHW Subjects

As presented in **Figure S1** for LHS, the LHS and COPDgene NHW subjects were stratified into Chronic Bronchitis (CB) or Emphysema (EM) sub-phenotypes based on clinical annotation parameters pertaining to chronic productive cough. For LHS, following quality-control analysis a total of 3,230 subjects were included. Of these, 527 were chronic bronchitis, 1,198 were emphysema, and 1,505 were controls. For COPDgene NHW there were a total of 5269 subjects, of which 556 were chronic bronchitis, 2223 were emphysema, and 2490 were controls.

Restriction of LHS GWAS SNP set

As presented in **Figure S1** the LHS GWAS SNP set was restricted to those within putative regulatory regions genes. We used Haploreg [13] to identify putative regulatory SNPs in the region spanning 5 kbp upstream of the transcript start site and 5 kbp downstream of transcript termination site of each gene. This SNP set was restricted to those within putative regulatory regions based on ENCODE annotation data [14]. This SNP set was further restricted to those associated with LHS CB subjects at threshold of p<0.05 {LHS CB Restricted} or with LHS EM subjects at threshold of p<0.05 {LHS EM Restricted}.

Integration of COPD-associated putative *cis*-rSNPs with GWAS

The COPD-associated putative BEC *cis*-rSNP set {BEC COPD DAE} was integrated with the {LHS CB Restricted} and {LHS EM Restricted} SNP sets (**Figure S2)**. SNPs that were significant (P<0.05) following correction for multiple testing were subjected to validation in sub-phenotyped COPDgene NHW subjects.

**Supplementary Figures**

**Figure S1. LHS Sub-phenotyping and SNP Restriction**


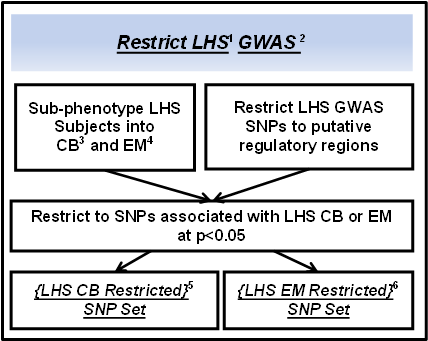


^1^LHS, Lung Health Study; ^2^GWAS: genome wide association study; ^3^CB: chronic bronchitis; ^4^EM: emphysema; ^5^(LHS CB Restricted): the set of SNPs restricted to putative regulatory regions and with p<0.05 for association with LHS CB subjects; ^6^(LHS EM Restricted) the set of SNPs restricted to putative regulatory regions and with p<0.05 for association with LHS EM subjects.

**Figure S2.** Integration of 35 targeted genes with {LHS CB Restricted} and {LHS EM Restricted} SNP sets, followed by test for validation in the COPDgene NHW CB and EM cohorts.


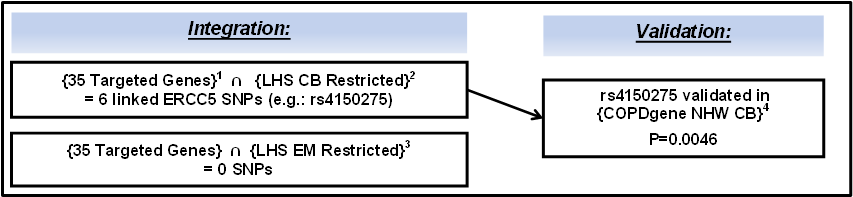


^1^ 35 Targeted genes.

^2^ (LHS CB Restricted): the set of SNPs restricted to putative regulatory regions and that have p<0.05 among LHS CB subjects;.

^3^{LHS EM Restricted}: the set of SNPs restricted to putative regulatory regions and that have p<0.05 among LHS EM subjects.

^4^ (COPDgene NHW CB): the set of SNPs in COPDgene NHW CB subjects.

**References**

1. Crawford EL, Levin A, Safi F, Lu M, Baugh A, Zhang X, Yeo J, Khuder SA, Boulos AM, Nana-Sinkam P *et al*: **Lung cancer risk test trial: study design, participant baseline characteristics, bronchoscopy safety, and establishment of a biospecimen repository**. *BMC pulmonary medicine* 2016, **16**:16.

2. Blomquist T, Crawford EL, Mullins D, Yoon Y, Hernandez DA, Khuder S, Ruppel PL, Peters E, Oldfield DJ, Austermiller B *et al*: **Pattern of Antioxidant and DNA Repair Gene Expression in Normal Airway Epithelium Associated with Lung Cancer Diagnosis**. *Cancer research* 2009, **69**(22):8629-8635.

3. Zhang X, Crawford EL, Blomquist TM, Khuder SA, Yeo J, Levin AM, Willey JC: **Haplotype and diplotype analyses of variation in ERCC5 transcription cis-regulation in normal bronchial epithelial cells**. *Physiol Genomics* 2016, **48**(7):537-543.

4. Sudmant PH, Rausch T, Gardner EJ, Handsaker RE, Abyzov A, Huddleston J, Zhang Y, Ye K, Jun G, Hsi-Yang Fritz M *et al*: **An integrated map of structural variation in 2,504 human genomes**. *Nature* 2015, **526**(7571):75-81.

5. Krjutskov K, Andreson R, Magi R, Nikopensius T, Khrunin A, Mihailov E, Tammekivi V, Sork H, Remm M, Metspalu A: **Development of a single tube 640-plex genotyping method for detection of nucleic acid variations on microarrays**. *Nucleic acids research* 2008, **36**(12):e75.

6. Blomquist TM, Crawford EL, Lovett JL, Yeo J, Stanoszek LM, Levin A, Li J, Lu M, Shi L, Muldrew K *et al*: **Targeted RNA-sequencing with competitive multiplex-PCR amplicon libraries**. *PloS one* 2013, **8**(11):e79120.

7. Blomquist T, Crawford EL, Yeo J, Zhang X, Willey JC: **Control for stochastic sampling variation and qualitative sequencing error in next generation sequencing**. *Biomol Detect Quantif* 2015, **5**:30-37.

8. Castel SE, Levy-Moonshine A, Mohammadi P, Banks E, Lappalainen T: **Tools and best practices for data processing in allelic expression analysis**. *Genome Biol* 2015, **16**:195.

9. Battle A, Brown CD, Engelhardt BE, Montgomery SB: **Genetic effects on gene expression across human tissues**. *Nature* 2017, **550**(7675):204-213.

10. Consortium GT: **The Genotype-Tissue Expression (GTEx) pilot analysis: Multitissue gene regulation in humans**. *Science (New York, NY)* 2015, **348**(6235):648-660.

11. Chang CC, Chow CC, Tellier LC, Vattikuti S, Purcell SM, Lee JJ: **Second-generation PLINK: rising to the challenge of larger and richer datasets**. *GigaScience* 2015, **4**:7.

12. Patterson N, Price AL, Reich D: **Population structure and eigenanalysis**. *PLoS genetics* 2006, **2**(12):e190.

13. Ward LD, Kellis M: **HaploReg: a resource for exploring chromatin states, conservation, and regulatory motif alterations within sets of genetically linked variants**. *Nucleic acids research* 2012, **40**(Database issue):D930-934.

14. Rosenbloom KR, Armstrong J, Barber GP, Casper J, Clawson H, Diekhans M, Dreszer TR, Fujita PA, Guruvadoo L, Haeussler M *et al*: **The UCSC Genome Browser database: 2015 update**. *Nucleic acids research* 2015, **43**(Database issue):D670-681.
